# Ubiquitin protein E3 ligase ASB9 suppresses proliferation and promotes apoptosis in human spermatogonial stem cell line by inducing HIF1AN degradation

**DOI:** 10.1186/s40659-023-00413-w

**Published:** 2023-01-23

**Authors:** Ning Li, Qianyin Zhou, Zhang Yi, Huan Zhang, Dai Zhou

**Affiliations:** 1 Operating Department of Xiangya Hospital, Central South University, Changsha, 410008 Hunan China; 2 Xiangya Nursing School, Central South University, Changsha, 410013 Hunan China; 3 Reproductive & Genetic Hospital of CITIC-Xiangya, Changsha, 410021 Hunan China; 4 Institute of Reproduction and Stem Cell Engineering, School of Basic Medicine Science, Central South University, Changsha, 410013 Hunan China; 5 College of Life Sciences, Hunan Normal University, Changsha, 410081 Hunan China; 6 Clinical Research Center for Reproduction and Genetics in Hunan Province, Changsha, 410021 Hunan China; 7 NHC Key Laboratory of Human Stem Cell and Reproductive Engineering, Central South University, Changsha, 410013 Hunan China

**Keywords:** Human, Testis, Spermatogonial stem cells, Proliferation, ASB9, HIF1AN

## Abstract

**Background:**

Spermatogonial stem cells (SSCs) are critical for sustaining spermatogenesis. Even though several regulators of SSC have been identified in rodents, the regulatory mechanism of SSC in humans has yet to be discovered.

**Methods:**

To explore the regulatory mechanisms of human SSCs, we analyzed publicly available human testicular single-cell sequencing data and found that Ankyrin repeat and SOCS box protein 9 (ASB9) is highly expressed in SSCs. We examined the expression localization of ASB9 using immunohistochemistry and overexpressed ASB9 in human SSC lines to explore its role in SSC proliferation and apoptosis. Meanwhile, we used immunoprecipitation to find the target protein of ASB9 and verified its functions. In addition, we examined the changes in the distribution of ASB9 in non-obstructive azoospermia (NOA) patients using Western blot and immunofluorescence.

**Results:**

The results of uniform manifold approximation and projection (UMAP) clustering and pseudotime analysis showed that ASB9 was highly expressed in SSCs, and its expression gradually increased during development. The immunohistochemical and dual-color immunofluorescence results displayed that ASB9 was mainly expressed in nonproliferating SSCs. Overexpression of ASB9 in the SSC line revealed significant inhibition of cell proliferation and increased apoptosis. We predicted the target proteins of ASB9 and verified that hypoxia-inducible factor 1-alpha inhibitor (HIF1AN), but not creatine kinase B-type (CKB), has a direct interaction with ASB9 in human SSC line using protein immunoprecipitation experiments. Subsequently, we re-expressed HIF1AN in ASB9 overexpressing cells and found that HIF1AN reversed the proliferative and apoptotic changes induced by ASB9 overexpression. In addition, we found that ABS9 was significantly downregulated in some NOA patients, implying a correlation between ASB9 dysregulation and impaired spermatogenesis.

**Conclusion:**

ASB9 is predominantly expressed in human SSCs, it affects the proliferation and apoptotic process of the SSC line through HIF1AN, and its abnormal expression may be associated with NOA.

**Supplementary Information:**

The online version contains supplementary material available at 10.1186/s40659-023-00413-w.

## Background

Infertility is becoming a global health problem, and an estimated incidence of 15%, with male factors accounting for about 50% of cases [1]. Azoospermia, particularly non-obstructive azoospermia (NOA), is a challenging male infertility condition to treat [2]. Aberrant spermatogenesis and spermatid maturation are the nature of NOA in humans. NOA can arise from failures at different stages of spermatogenesis, from germinal stem cells to sperm formation. Owing to the complexity of spermatogenesis and testicular function, and the high degree of genetic heterogeneity, effective treatments for NOA are still lacking. It is also a challenge in the field of male reproduction [3].

In recent years, stem cell regenerative medicine has advanced rapidly, allowing the use of germinal stem cells to restore abnormal testicular function caused by germ cells. Spermatogonial stem cells (SSCs) continuously self-renew and differentiate into mature spermatozoa hence are crucial to spermatogenesis [4, 5]. In 1994, Brinster et al. successfully transplanted spermatogonia to restore spermatogenesis in the testes of recipient mice [6]. Long-term cultivation and transplantation of mouse SSCs is now a well-established technology [7, 8]. However, the mouse in vitro culture technique is unsuitable for human SSCs, and insufficient self-renewal and proliferation are the primary issues with in vitro-cultured human SSCs [9]. Therefore, understanding human SSC self-renewal and proliferation is the key to achieving long-term in vitro culture and the use of SSC to treat male infertility.

Glial cell line-derived neurotrophic factor (GDNF) is a vital growth factor required for the self-renewal of SSCs [7]. GDNF binds to the Gfra1/c-Ret receptor, activates the RAS/ERK [10], PI3K/AKT, and MAPK signaling pathways [11], and regulates the expression of downstream *Etv5*, *Bcl6b*, *Lhx1*, *Brachyury*, and *Cxcr4* genes to maintain the self-renewal of SSCs [12]. FGF2 [13], FGF5 [14], and FGF9 [15] promote the proliferation of SSCs via the ERK and MAPK pathways. However, the roles of FGFs on SSCs differ from that of GDNF [16], and FGF2 expands differentiation-prone spermatogonia, suggesting that it may contribute to early differentiation decisions of SSCs [17]. In addition, endogenous molecules participate in the maintenance of spermatogonial stem cell self-renewal. PLZF [18, 19], FOXO1 [20], CARF [21], and PRMT5 [22] play their respective roles in the process of SSC self-renewal, and deletion of either molecule results in the gradual loss of SSC, ultimately leading to infertility. Retinoic acid (RA) is a critical molecule in the stimulation of SSC differentiation [23, 24]. Ngn3, Sox3, and Lin28a expression are significantly upregulated in differentiated cells [25], while Ngn3^+^ progenitors expressed more RARγ receptors and were more sensitive to RA stimulation [26]. Wnt/β-catenin signaling promotes the transition of spermatogonial stem cells from a self-renewing state to an RA signaling-sensitive state. Shisa6 can block cell differentiation by inhibiting WNT signaling [27].

The regulatory mechanisms of SSCs have been better understood by studying mouse models. However, due to species differences, sources of human testicular tissue, and other factors, many research results on mouse SSCs do not apply to humans, and the regulatory mechanisms of human SSCs are still poorly understood. Human and mouse spermatogonial stem cells differ in cell types and biochemical characteristics, indicating that their regulatory mechanisms may be distinct. For example, A_s_ spermatogonia are considered SSCs in mice, whereas A_dark_ and A_pale_ spermatogonia are considered human spermatogonial stem cells [28]. The seminiferous epithelium of mice can be divided into 12 spermatogenic cycles, whereas humans are typically divided into 6 [29]. Oct4 is a marker molecule for rodent spermatogonial stem cells [30], but Oct4 is not expressed in human spermatogonial stem cells [31].

Only a few human studies on SSC proliferation and apoptosis have been reported. PAK1 promotes human SSC proliferation via signaling pathways such as PDK1/KDR/ZNF367 [32]. MiR-1908-3p promotes human SSC proliferation by targeting and degrading KLF2 mRNA [33], while CARF influences the self-renewal and proliferation of both human and mouse SSCs via the WNT signaling pathway [21]. In addition, we have previously demonstrated that the transcription factor TCF3 enhances SSC proliferation and inhibits apoptosis by promoting the transcription of genes such as PODXL [34]. However, the developmental regulatory mechanisms of human spermatogonial stem cells require additional research.

To investigate the regulatory mechanism of human SSC, we performed an integrated analysis of human testis single-cell sequencing data from GSE109037 [35] and GSE120508 [36] and identified several genes highly expressed in human SSC, including *ASB9, C19orf84*, *CST3,* and *GNB2L1*. Immunohistochemical results indicated that ASB9 is predominantly expressed in human SSCs, suggesting that it may influence the fate of SSCs [37]. ASB9 was reported to influence cell functions by regulating the ubiquitination and degradation of proteins such as CKB [37], HIF1AN [38], CKM [39], and CKMT1B [40]. However, little is known about its role in SSCs.

Our study demonstrated that ASB9 was predominantly localized in human SSCs and that ASB9 overexpression significantly inhibited proliferation and promoted apoptosis in a human SSC-derived cell line. Based on the results of protein co-immunoprecipitation, HIF1AN had direct interaction with ASB9, and re-expression of HIF1AN partially restored the cell proliferation and apoptotic changes caused by ASB9 deficiency. In addition, ASB9 levels were significantly down-regulated in human testes with impaired spermatogonia and spermatocyte maturation, indicating that ASB9 may be associated with abnormal spermatogenesis in humans. These findings reveal new insights into the developmental regulation of SSC in humans and provide ideas for male infertility diagnosis and treatment.

## Materials and methods

### Collection of human testis tissue

The Ethical Committee of the Reproductive & Genetic Hospital of CITIC-Xiangya, Basic Medical Science School, Central South University, approved this study (LL-SC-2021–025). All participants were required to sign informed consent. Twenty patients (5 OA and 15 NOA) between the ages of 25 and 46 who underwent microdissection testicular sperm extraction (m-TESE) provided approximately 20 mg of testicular tissues for this study. Samples were washed thrice with PBS containing 1% streptomycin and penicillin, fixed in 4% paraformaldehyde (PFA), or stored in liquid nitrogen.

### Single-cell RNA-sequencing data analysis

Six normal adult testis single cell RNA sequencing (scRNA-seq) datasets, three from GSE109037 and three from GSE120508, were analyzed by the Seurat program (http://satijalab.org/seurat/, R package, version 3.0). First, expression matrix data were imported into R using the Read.table or Read.csv function, and Seurat objects were developed from every assay. After filtering each assay, they were normalized using the default settings. Only cells expressing > 500 genes and with < 20% of reads mapped to the mitochondrial genome were retained. Variable features for each object were identified and all data merged using the IntegrateData function. After deleting mitochondrial and ribosomal genes, UMAP and clustering analysis was conducted using the top 2500 highly variable genes and top 20 principal components (PCs) on the combined dataset, the samples are projected into 2 dimensions and presented with a scatter plot. Next, a more in-depth SSCs clustering identified two subclusters. Starting with the subcluster at State 0, SSCs from the UMAP plot were subjected to pseudotime assessment via “Monocle” in R (https://cole-trapnell-lab.github.io/monocle3/, version 3.0). Heatmaps were generated in pseudotime order, with line plotting done and curve fitting using the ‘‘auto’’ method of “ggplot2” in R (https://ggplot2.tidyverse.org/).

### Culture of immortalized human SSC line

To establish the human SSC line, the human SV40 large T antigen was overexpressed in human primary GPR125-positive undifferentiated spermatogonia [41]. Immortalized human SSCs retained many primary cell properties and were positive for several SSC markers, such as PLZF, GFRA1, UCHL1, and THY1 [34]. Cultures of the human SSC line were done in DMEM/F12 (Gibco, Grand Island, NY) with 10% FBS (Gibco) and 100 unit/mL streptomycin/penicillin (Invitrogen, CA, USA) at 34℃ in a 5% CO_2_ atmosphere. Every 3 days, cells were passaged using 0.53 mM EDTA and 0.05% trypsin (Invitrogen).

### Immunohistochemistry and immunofluorescence assays

For immunohistochemistry, testis sections were deparaffinized using xylene. Graded ethanol was used for rehydration. Heat-induced antigen retrieval (HIER) was performed at 98 °C for 18 min in 0.01 M sodium citrate buffer in a beaker, and 3% H2O2 (Zsbio, Beijing, China) was used to inhibit endogenous peroxidase activities. Then, cell sections were permeabilized using 0.25% Triton X-100 (Sigma) for 15 min, followed by 1 h of blocking with5% BSA at room temperature, overnight incubation with primary antibodies (Additional file 1: Table S1) at 4 °C and rinsed in PBS. They were incubated for 1 h with HRP-labeled secondary antibody at room temperature. The 3,3′-diaminobenzidine (DAB) chromogen kit (Dako, Glostrup, Denmark) was used for chromogen detection. Finally, sections were hematoxylin stained. For immunofluorescence, sections were incubated with a second antibody conjugated to Alexa Fluor for 1 h at room temperature. The rest of the tissue can be used as a control of non-specific binding for the primary and secondary antibodies used. Nuclei were 4′,6-diamidino2-phenylindole (DAPI) stained and imaged using a Zeiss microscope.

### Western blot and immunoprecipitation assays

Cells and testicular samples were ground and lysed for 30 min on ice using the Radioimmunoprecipitation assay (RIPA) lysis buffer (Thermo Scientific), after which they were centrifuged at 12,000 g to obtain clear lysates. Protein concentrations in lysates were determined by the BCA kit (Thermo Scientific). The cell lysate was treated with control rabbit IgG or primary antibodies and incubated overnight at 4 °C. The following day, Protein G magnetic beads were added to the supernatants and incubated for 2 h at 4 °C. Samples were washed thrice using a washing buffer, after which beads were magnetically pelleted, re-suspended, and boiled for 5 min at 95 °C. For every sample, total protein extracts (30 µg) were subjected to SDS-PAGE (Bio-Rad), and western blotting was conducted according to a previous protocol [42]. The antibodies used in this assay and their dilution ratios are shown in Additional file 1: Table S1. Intensities of immunoreactive protein bands were visualized using Chemiluminescence (Bio-Rad).

### Plasmids transfection

The flag-ASB9 and flag-HIF1AN overexpression plasmids (Additional file 1: Fig S1) were prepared by SinoBiological (Beijing, China). The pCMV3-flag plasmid without targeting sequences was the negative control (NC). Transfection of cells with 2.5 μg plasmids was done using the Lipofectamine 3000 transfection system (Cat No, Life Technologies, CA, USA), as instructed by the manufacturer. For double-transfections, 1.25 μg of each plasmid was used. Transfection efficiency was approximately 70% determined by dual transfection of GFP reporter plasmids. After 48 h of transfection, cells were obtained to determine alterations in protein and gene expressions.

### CCK-8 assay

After plasmid transfection, the proliferative potential of human SSC line was evaluated using the CCK-8 assay kit (Dojindo, Kumamoto, Japan), according to the manufacturer's instructions. The cell culture media was substituted with 10% CCK-8 reagents, incubated for 3 h, and optical density (OD) values were measured at 450 nm using a microplate reader (Thermo Scientific).

### EdU incorporation assay

5000 human SSC lines/well were seeded in a 96-well plate containing DMEM/F12 media with 50 μM EdU (RiboBio) and incubated for 12 h. Cells were rinsed using DMEM and then fixed in 4% PFA. After that, they were neutralized using glycine (2 mg/mL) and then permeabilized for 10 min using Triton X-100 (0.5%) at room temperature (RT). EdU immunostaining was conducted using the Apollo staining reaction buffer. Cell nuclei were stained using Hoechst 33,342. Percentages of EdU-positive cells were determined by counting at least 500 cells. Fluorescence microscopy (Zeiss) was used for imaging.

### Annexin V-APC/PI staining and flow cytometry

To evaluate the apoptosis of the human SSC line after affected by the ASB9 overexpression plasmid, cells were digested and rinsed twice in ice-cold PBS. Then, 10 [6] cells were re-suspended in Annexin V Binding Buffer (BD Biosciences, NJ, USA), incubated in the presence of Annexin V labeled APC (5 µl) and 10 µL of PI solution for 15 min at RT away from light. Cells were analyzed using a C6 flow cytometry instrument (BD Biosciences).

### TUNEL assay

The In Situ Cell Death Detection Kit (Roche) was used to examine the apoptosis of the human SSC line affected by plasmids. Treatment of cells with proteinase K (20 mg/ mL) was done for 15 min at RT, followed by incubation for 1 h with dUTP labeling/terminal deoxynucleotidyl transferase (TdT) enzyme buffer away from light. The cell nuclei were counterstained with DAPI. At least 500 cells per sample were assessed by a Zeiss fluorescent microscope.

### Statistical analysis

GraphPad Prism 8.0 (GraphPad Software, CA, USA) was used for analyses. Values are shown as mean ± SD for n = 3. The *t*-test or Wilcoxon matched-pairs signed rank test was used to determine differences between the groups according to the data distribution, and *p* < 0.05 was statistically significant.

## Results

### Human spermatogonia stem cell profiling based on scRNA-seq analysis

**Fig. 1:**
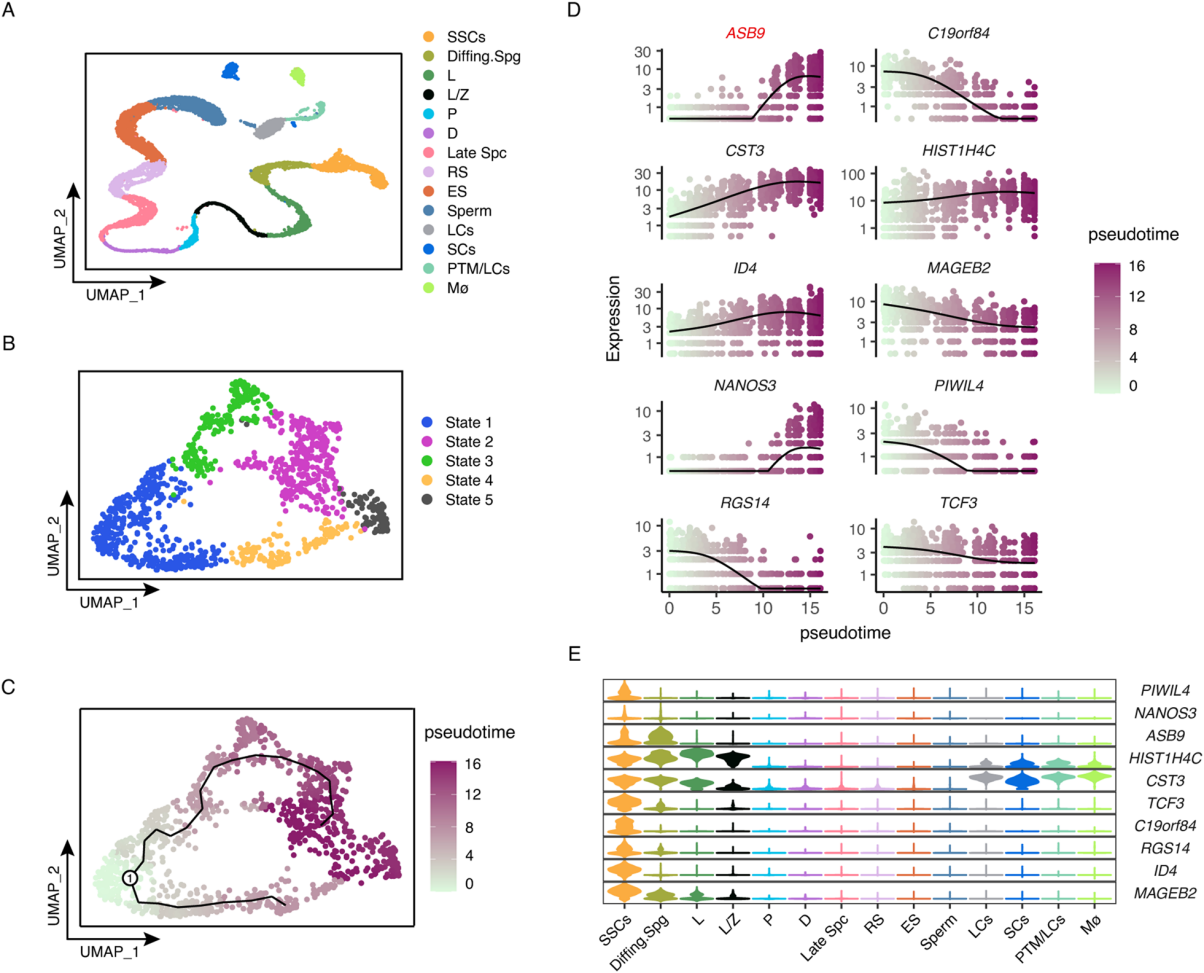
Human spermatogonia stem cell profiling based on scRNA-seq analysis. **A** UMAP and clustering analyses of testis single-cell transcriptomic data from GSE109037 and GSE120508. Each dot represents a single cell and is colored according to the identity of the cluster in the figure. **B** Reclustering analysis of SSCs cluster, SSCs were subgrouped into five stages and colored according to the legend. **C** Pseudo-time analysis on SSCs. The black line in the figure shows the cell developmental trajectory, and character 1 represents the starting point of the developmental trajectory. **D** Pseudo-time analysis on Top 10 differentially expressed genes at different stages. The x-axis denotes pseudo-time (defined in **D**). and the y-axis represents gene expression levels. The black curves in the graph showed the average expression level of the specified gene in all SSCs accompanying the developmental trajectory. **E** The Violin plot showed the expression pattern of the Top 10 differentially expressed genes in all testicular cells. UMAP: uniform manifold approximation and projection, SSCs: spermatogonial stem cells, Diff.ing Spg: differentiating spermatogonia, L: leptotene spermatocytes, L/Z: leptotene/zygotene spermatocytes, P: pachytene, D: diplotene spermatocytes, Late Spc: late spermatocytes, RS: round spermatids, ES: elongated spermatids, LCs: Leydig cells, SCs: Sertoli cells, PTM/LCs: peritubular myoid cells/Leydig cells, Mø: macrophages

To explore the mechanisms regulating human SSCs, adult testis scRNA-seq datasets, including GSE109037 [35] and GSE120508 [36], were analyzed. All cells were divided into 14 clusters using the Seurat package on R. The levels of common testicular cell marker genes, including SSCs markers (*ID4*), differentiating markers (*KIT* and *STRA8*), meiosis markers (*SYCP3*, *SPO11*, *OVOL2*, and *NME8*), spermatid structure proteins (*TNP2* and *PRM2*) and some somatic markers were evaluated to identify the respective cell types. The 14 cell populations included spermatogonial stem cells (SSCs), differentiating spermatogonia (Diffing. Spg), leptotene spermatocytes (L), leptotene/zygotene spermatocytes (L/Z), diplotene spermatocytes (D), pachytene spermatocytes (P), late spermatocytes (Late Spc), round spermatids (RS), elongated spermatids (ES), sperm, Leydig cells (LCs), Sertoli cells (SCs), peritubular myoid cells/Leydig cells (PTM/LCs), and macrophages (Mø) (Fig. 1A). To further examine the fate regulation of SSCs, we extracted data on SSCs clusters for in-depth analysis and subdivided the SSC into 5 clusters (Fig. 1B). Using the monocle package on R, a quasi-chronological analysis of cells was performed to determine their developmental trajectories. It was discovered that, based on the expression levels of undifferentiated spermatogonia marker genes (*PIWIL4* and *NANOS3*) [43], State 1 was the developmental starting point, State 2, 3, and 4 were transitional periods of development, and State 5 was late development (Fig. 1C). Differential gene expression analysis identified several genes, including *ASB9*, *C19orf84*, and *CST3* (Fig. 1D). Using violin plots, we evaluated the distribution of these genes in all testicular cells (Fig. 1E). Among these differentially expressed genes, *ASB9* expression gradually increased during SSC development and is abundant in Diffing. Spg. This implies that *ASB9* may be associated with the fate regulation of SSCs.

### ASB9 is expressed mainly in human spermatogonia stem cells

**Fig. 2:**
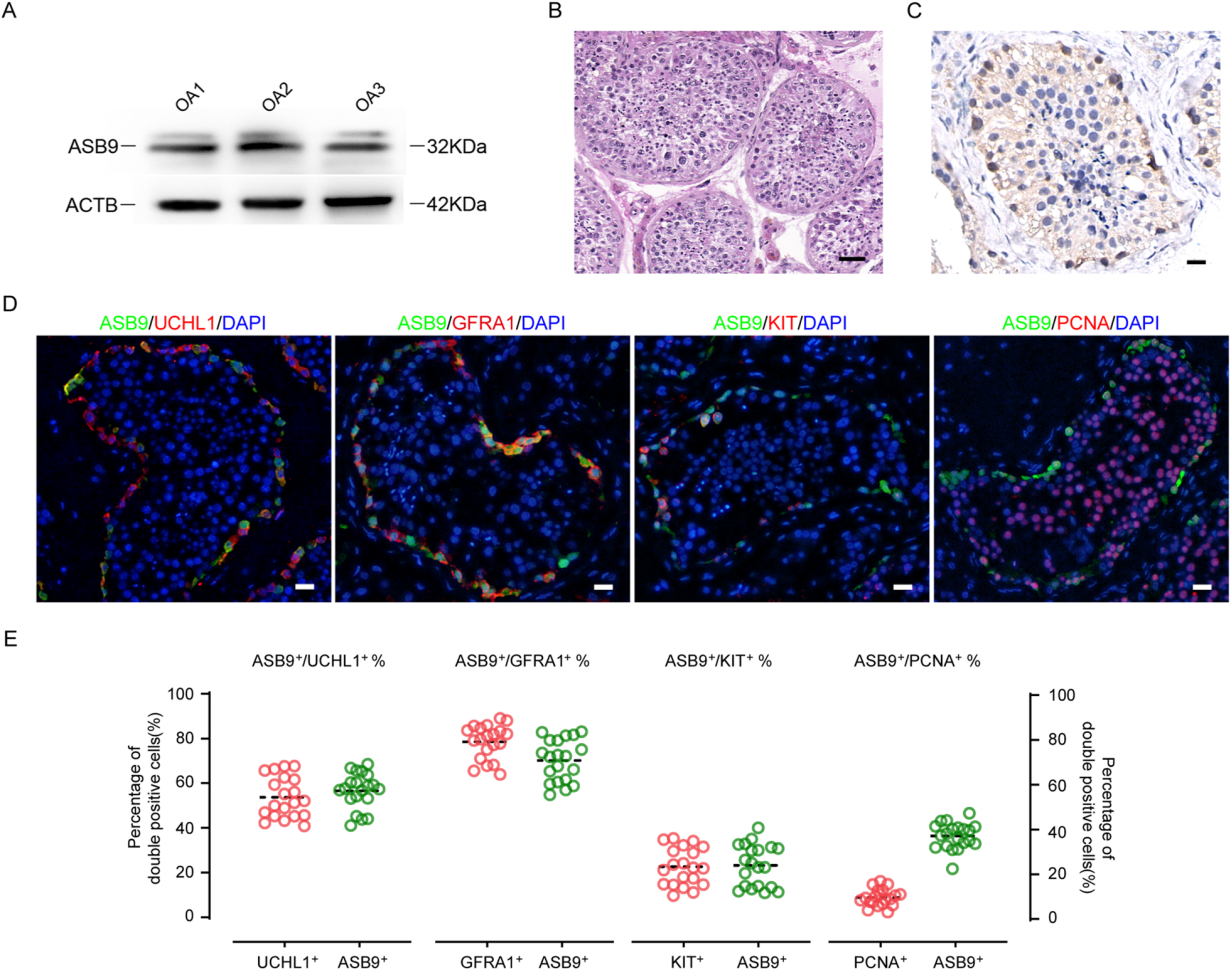
The expression of ASB9 in human testis. **A** Western blot showed the level of ASB9 in three testes of OA samples with normal spermatogenesis, ACTB was used as the loading control of total protein. **B** Representative H&E image of OA testicular tissues. Scale bars,200 μm. **C** Immunohistochemistry images for cell localization of ASB9 in OA sample. Scale bars, 50 μm. **D** Double immunostaining revealing co-expressions of ASB9 with UCHL1, GFRA1, KIT, and PCNA in human testis with normal spermatogenesis. Scale bars, 50 μm. **E** Percentages of ASB9^+^ cells co-expressing UCHL1, GFRA1, PCNA, and KIT. At least 20 seminiferous tubules were assessed. Each circle represents a count result, and the dashed line in the figure shows the mean value of the 20 count results

To authenticate the scRNA-seq findings, we examined protein levels of ASB9 in human testicular tissues. The ASB9 protein levels in testicular samples from three OA patients with normal spermatogenesis were analyzed by Western blot (Fig. 2A). Immunohistochemical analysis of normal testis revealed that ASB9 is mainly localized in cytoplasms of cells adjacent to seminiferous tubule basement membranes, implying its expressions in spermatogonia (Fig. 2B, C). Therefore, we further analyzed the cell subtypes in which ASB9 was expressed using double immunofluorescence (Fig. 2D). ASB9-positive cells in at least 20 seminiferous tubule sections were counted, and the results showed that ASB9 was co-expressed with UCHL1 in 56.56% ± 7.99% of the cells, and 71.03% ± 9.34% ASB9-positive cells expressed GFRA1 (a marker of spermatogonial stem cells). In addition, 24.01% ± 9.28% of ASB9-positive cells expressed KIT, a marker for differentiating spermatogonia. It is important to highlight that only 37.10% ± 5.77% of ASB9-positive cells expressed PCNA, a marker of proliferating cells (Fig. 2D, E). These data validated the bioinformatics results that ASB9 was primarily expressed in human SSC and that it may influence human SSC proliferation.

### ASB9 overexpression inhibits the proliferation of human spermatogonia stem cell lines

**Fig. 3:**
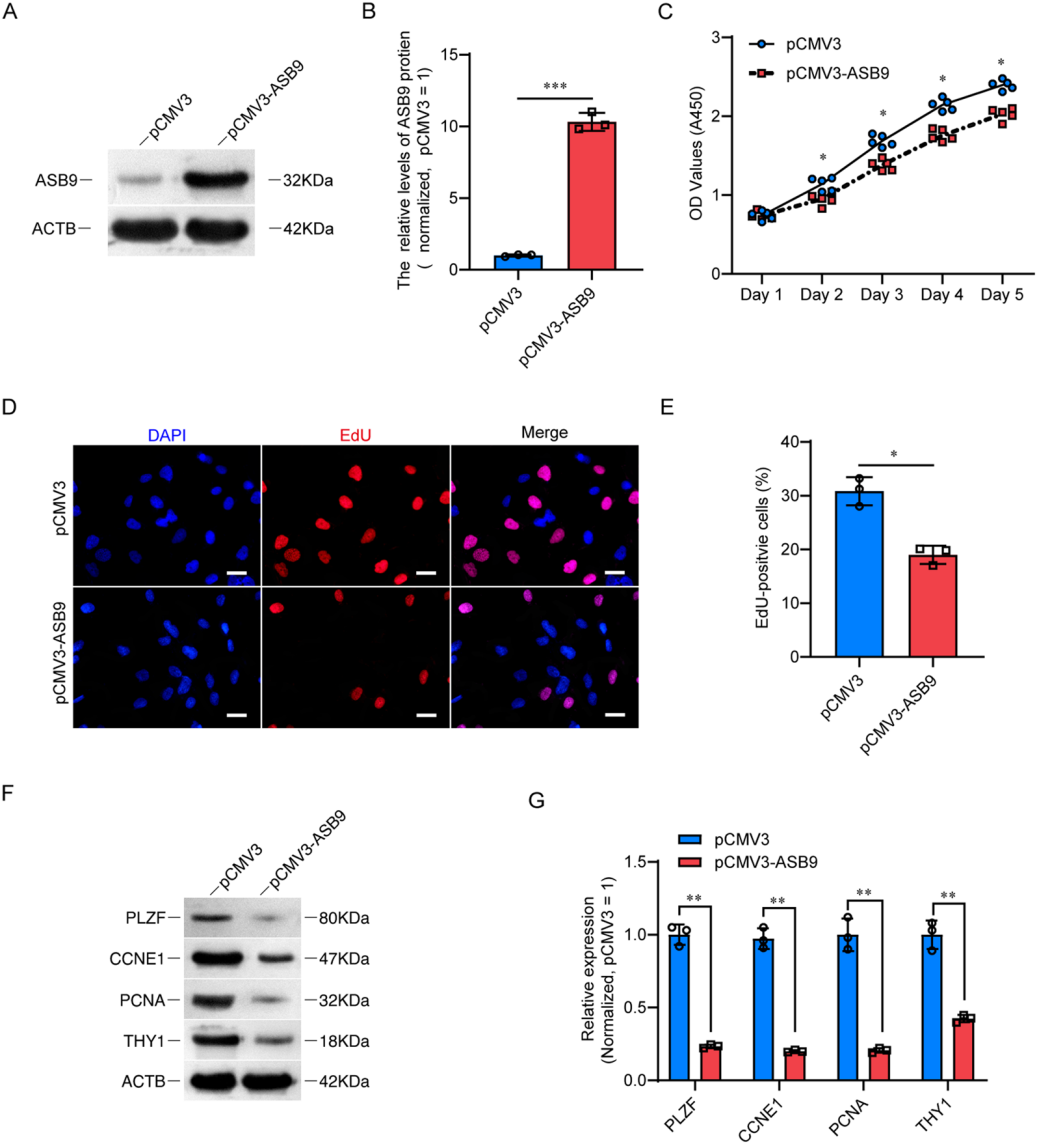
The influences of ASB9 overexpression on human SSC line proliferation. **A**, **B** Western blot showed the level of ASB9 in the human SSC line after transfected with pCMV3 and pCMV3-ASB9 plasmid. **C** CCK-8 assay displayed the proliferation of human SSC line transfected with pCMV3 and pCMV3-ASB9 plasmid. **D**, **E** Percentages of EdU-positive cells after transfection of pCMV3 and pCMV3-ASB9 plasmid. **F**, **G** Western blot showed the protein level changes of PLZF, CCNE1, PCNA, and THY1 after transfection with a pCMV3-ASB9 plasmid, ACTB protein served as the loading control. Scale bars in D, 50 μm. *Indicates significant difference at *p* < 0.05, ** indicates significant difference at *p* < 0.01, *** Indicates significant difference at *p* < 0.001

To elucidate the significance of ASB9 in the proliferation of human SSC, immortalized human SSC lines were utilized. We constructed a plasmid for ASB9 expression (pCMV3-Flag-ASB9) and transfected it into a human SSC line. Western blot results showed that the protein level of ASB9 was significantly up-regulated after transfection, confirming the effectiveness of transfection (Fig. 3A, B). Subsequently, we examined the proliferation of cells after ASB9 overexpression using the CCK8 assay, the results demonstrated that ASB9 upregulation significantly inhibited the proliferation of SSC line from day 2 to day 5 (Fig. 3C). This result was also supported by EdU assay, the percentage of EdU-positive cells was reduced in the ASB9 overexpression group, relative to the negative control (NC) group (18.98% ± 1.71% vs. 30.83% ± 2.62%, p < 0.05) (Fig. 3D, E), suggesting that cellular DNA synthesis was impaired following ASB9 overexpression. Similarly, we examined the levels of several proteins associated with SSC proliferation, including PLZF, CCNE1, PCNA, and THY1, the levels of these proteins were downregulated upon transfection with ASB9 expression plasmids (Fig. 3F, G), implying that cell proliferation and self-renewal were repressed. These results suggested that ASB9 inhibits the proliferation of human SSC line.

### ASB9 upregulation induces apoptosis in spermatogonia stem cell line

**Fig. 4:**
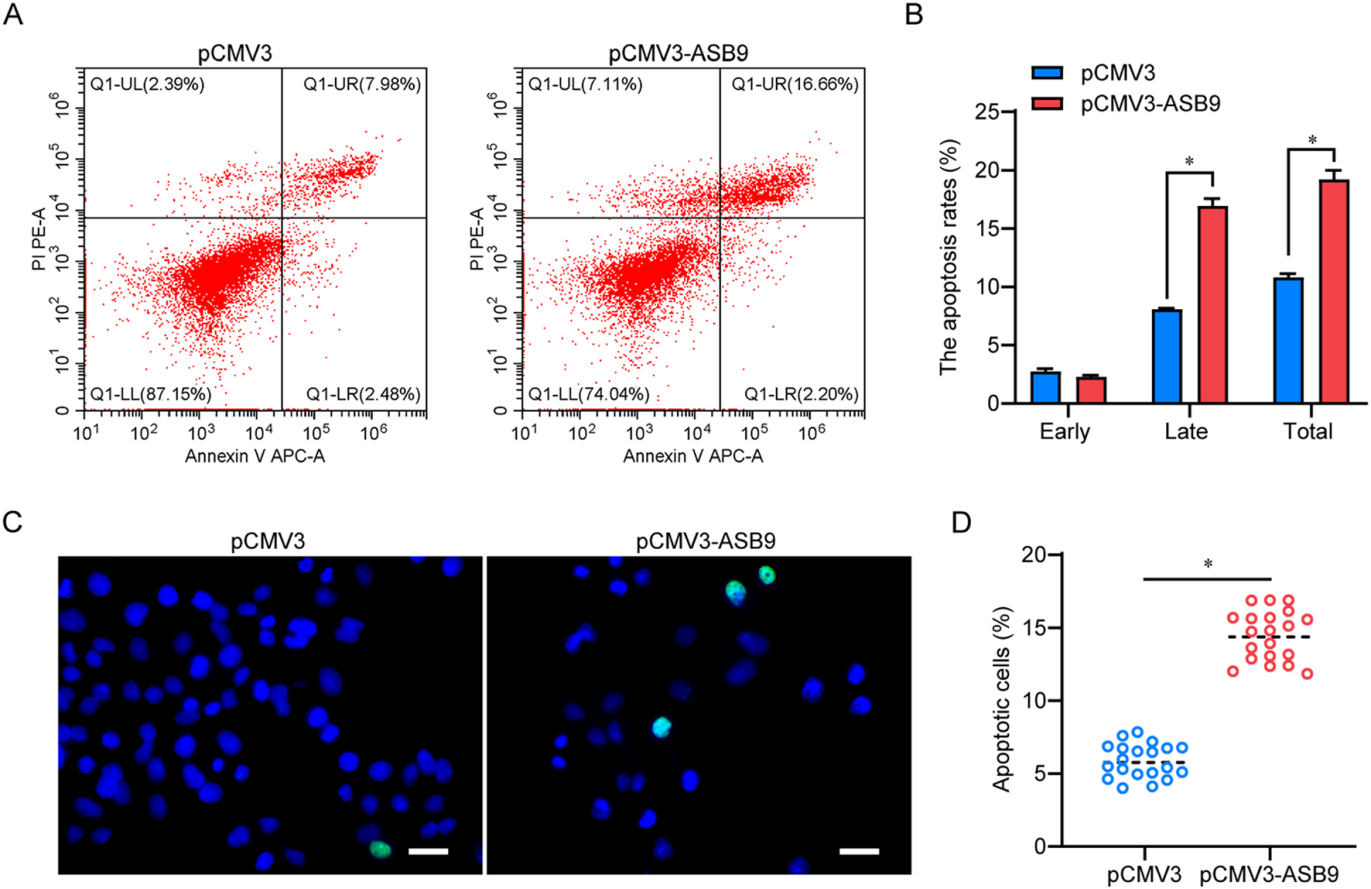
The effects of ASB9 overexpression on human SSC line apoptosis. **A**, **B** Flow cytometry and APC Annexin V analysis displayed early and late apoptotic cell percentages in human SSC lines transfected with pCMV3 and pCMV3-ASB9 plasmid. **C**, **D** TUNEL analysis showed the proportions of TUNEL^+^ cells in the human SSC line transfected with pCMV3 and pCMV3-ASB9 plasmid. * *p* < 0.05 indicates significant differences between pCMV3 and pCMV3-ASB9 groups

After ASB9 overexpression in human SSC line, a significant increase in suspended cells and debris was observed. Therefore, we examined the apoptotic changes of the cells using Annexin V/PI staining and flow cytometry, which showed that enforced expression of ASB9 significantly promoted late apoptosis of human SSC line relative to cells transfected with pCMV3-Flag (early apoptosis: 2.28% ± 0.15% vs. 2.74% ± 0.26%, *p* < 0.05; late apoptosis: 8.07% ± 0.09% vs. 16.94% ± 0.63%, *p* < 0.05) (Fig. 4A, B). The TUNEL assay yielded similar findings, with a marked increase in the rate of cellular DNA fragmentation (5.87% ± 1.16% vs. 14.47% ± 1.71%, *p* < 0.05) (Fig. 4C, D). Thus, ASB9 overexpression promotes apoptosis in the human SSC line.

### Screening of ASB9 downstream target proteins

**Fig. 5:**
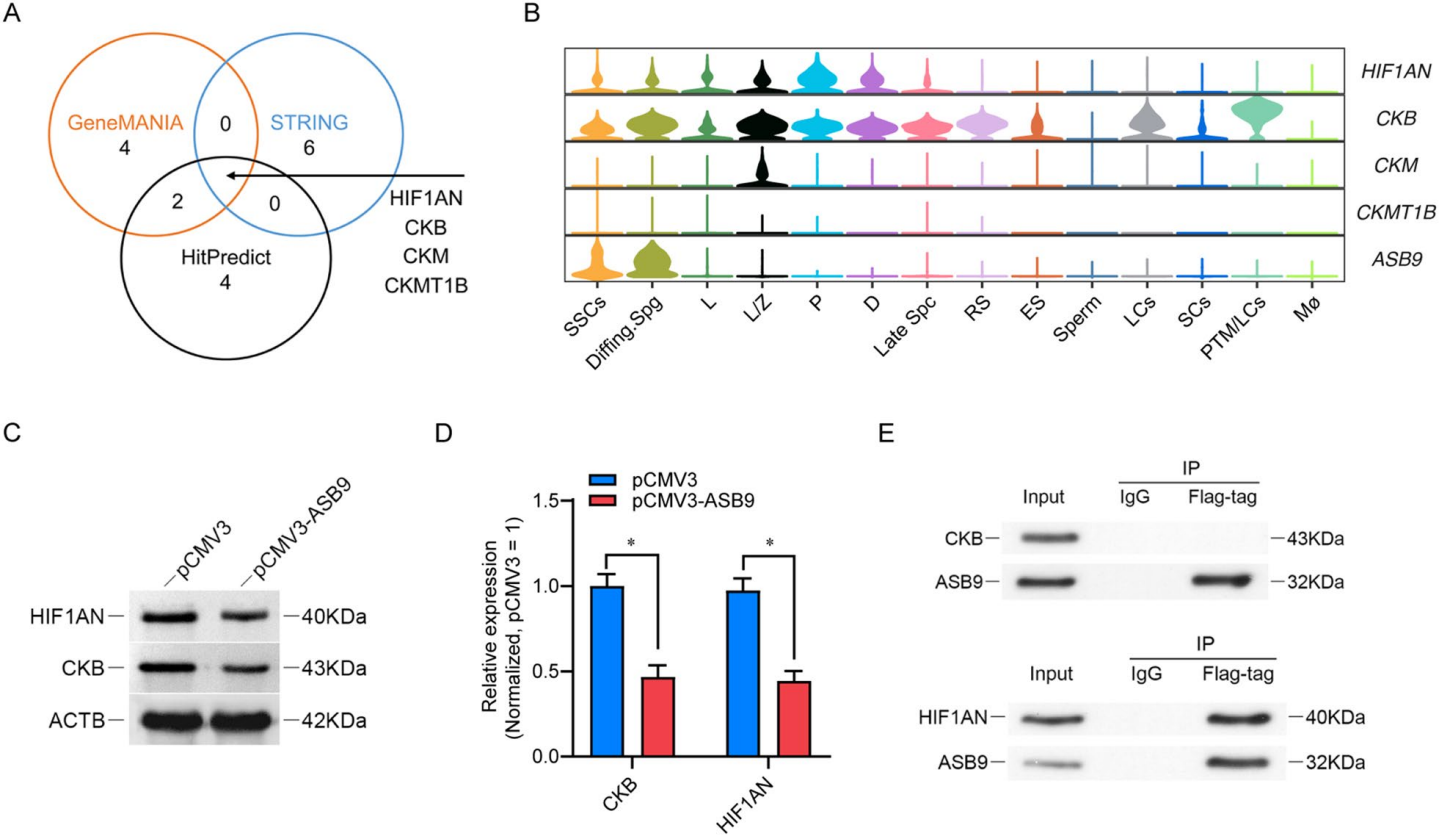
ASB9 downregulated HIF1AN in human SSC line. **A** Prediction of ASB9-interacting proteins by GeneMANIA, STRING and HitPredict. **B** The Violin plot showed the expression of HIF1AN, CKB, CKM and CKMT1B in testicular cells. **C**, **D** Western blot revealed that ASB9 overexpression led to a reduction of HIF1AN and CKB. **E** Immunoprecipitation demonstrates the interaction of ASB9 with HIF1AN. * *p* < 0.05 indicates significant differences between pCMV3 and pCMV3-ASB9 groups

ASB9 has been identified as a ubiquitin E3 ligase that affects cellular function by regulating the ubiquitination and degradation of certain proteins [37]. We used the database of interacting proteins, including GeneMANIA, STRING, and HitPredict, to predict the targets of in SSC line. CKB, CKM, HIF1AN, and CKMT1B were identified in the prediction results of the three databases (Fig. 5A). scRNA-seq analysis revealed that human SSC highly expressed CKB and HIF1AN (Fig. 5B), implying that ASB9 may have an interaction with them. Therefore, we further investigated the effects of ASB9 overexpression on CKB and HIF1A levels. Western blot showed that elevated ASB9 markedly downregulated the level of CKB and HIF1AN (Fig. 5C, D). However, protein immunoprecipitation experiments revealed that HIF1AN, not CKB, interacted with ASB9 (Fig. 5E). These findings imply that HIF1AN may be a potential target of ASB9.

### HIF1AN is responsible for ASB9-mediated proliferation depression of the SSC line

**Fig. 6:**
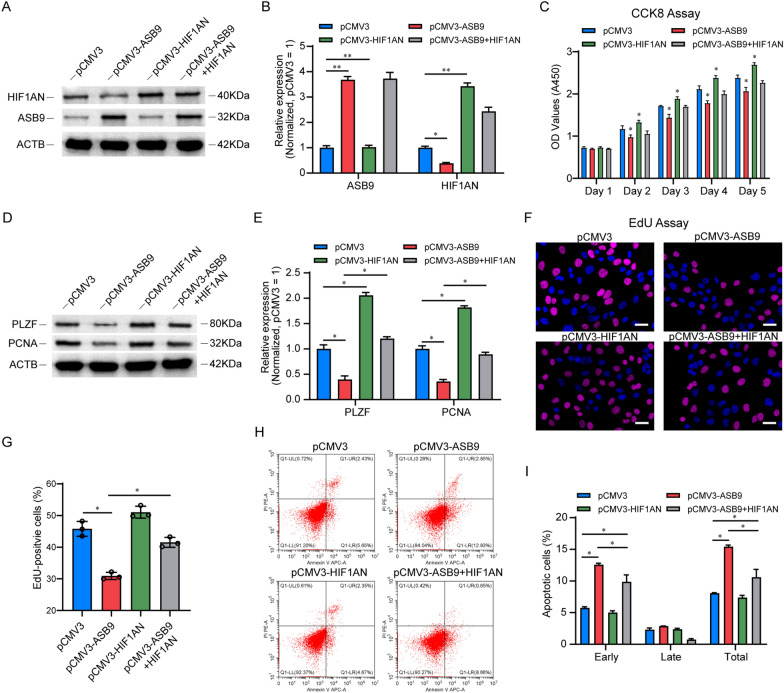
ASB9 inhibited SSC proliferation by regulating HIF1AN. **A**, **B** Western blot analysis was performed to confirm transfection efficiency, and the results showed that HIF1AN expression plasmid re-expressed HIF1AN protein in ASB9 overexpression SSCs. **C** CCK8 was used to detect cell proliferation after HIF1AN was re-expressed in ASB9 overexpression SSCs. **D**, **E** Western blot showed the protein level of PLZF and PCNA after transfection with HIF1AN expression plasmid in ASB9 overexpression SSCs. (F-G) Cellular DNA synthesis was detected by EdU analysis. Scale bar, 20 μm. (H-I) Cell apoptosis analysis after re-expressed HIF1AN using Flow Cytometry. * indicates significant difference (*p* < 0.05) and ** indicates extremely significant difference (*p* < 0.01)

To establish the significance of HIF1AN in ASB9-induced inhibition of cell proliferation, we simultaneously transfected expression plasmids for ASB9 and HIF1AN in SSC line and examined protein levels using Western blot (Fig. 6A, B). CCK8 experiments showed that elevated levels of HIF1AN promoted cell proliferation and reversed the proliferation inhibition caused by ASB9 overexpression (Fig. 6C). The re-expression of HIF1AN also significantly elevated the levels of SSC proliferation-associated proteins PLZF and PCNA (Fig. 6D, E). EdU assay results indicated that HIF1AN upregulation could partially restore the decrease of DNA synthesis in SSC line caused by ASB9 overexpression (Fig. 6F, G). We also examined the role of HIF1AN in the apoptotic process, and the results of flow cytometry showed that HIF1AN counteracted the increase of apoptosis caused by ASB9 upregulation (Fig. 6H, I). These results demonstrated that HIF1AN overexpression significantly antagonized the changes in cell proliferation and apoptosis induced by ASB9 upregulation, indicating that HIF1AN is a functional target of ASB9 in SSC line.

### ASB9 deficiency may be associated with non-obstructive azoospermia in humans

**Fig. 7:**
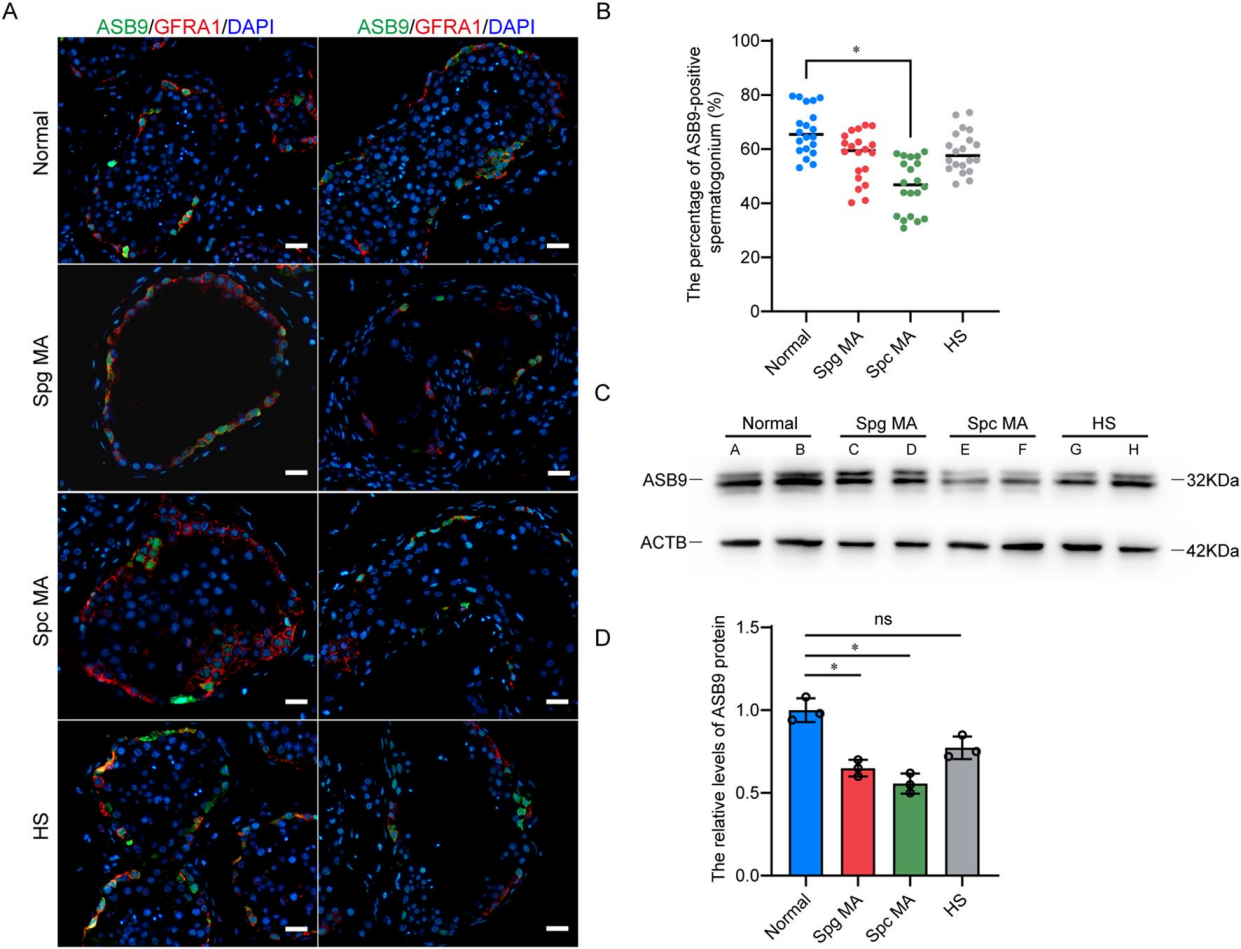
ASB9 expressions in testicular tissues from OA and NOA patients. **A**, **B** Percentages of GFRA1-positive SSCs (red) with ASB9 expression (green) in OA and NOA samples. **C**, **D**) Western blot results showed ASB9 protein levels in OA and NOA patients. In **A**, each picture represents one testis sample. Each group had two samples. Spg MA: spermatogonium maturation arrest. Spc MA: spermatocyte maturation arrest, HS: hypospermatogenesis. Scale bars in A, 50 μm. * *p* < 0.05 indicates significant differences between OA patients with normal spermatogenesis and NOA patients

In clinical practice, non-obstructive azoospermia (NOA) is a severe male infertility disease. Based on the findings of pathological testicular tissue, NOA can be grouped into spermatogonia maturation arrest (Spg MA), spermatocyte maturation arrest (Spc MA), spermatid maturation arrest (Std MA), hypo-spermatogenesis (HS), and Sertoli cell-only syndrome (SCOS). To determine the associations between ASB9 and impaired spermatogenesis in adults, the levels and distribution of ASB9 were determined in eight adult testes (Additional file 1: Fig S2). Using immunofluorescence co-localization with GFRA1 to detect changes in the distribution of ASB9, we found that the percentage of ASB9-positive spermatogonial stem cells was significantly decreased in Spc MA tissues, and we did not observe the translocation of ASB9 in cells (Fig. 7A, B). Western blotting results indicated that ASB9 levels were decreased in all patients with abnormal spermatogenesis, but this reduction was only significant in Spc MA and Spg MA samples (Fig. 7C, D). Taken together, we believe that the decrease in ASB9 may be associated with spermatogenesis disorders in humans, but additional evidence is required.

## Discussion

SSCs are responsible for long-term spermatogenesis by balancing self-renewal and differentiation [4]. Despite discovering several regulatory mechanisms in mice SSC, the regulation of SSC in humans is still poorly understood due to limited sample sources and species differences between humans and mice. Insufficient proliferation viability of human SSC in vitro is a significant obstacle to our study and application [9]. Therefore, exploring the mechanisms of SSC self-renewal and proliferation in humans is the key to resolving this problem. scRNA sequencing has provided us with a transcriptional map of human SSC and led to the discovery of a significant number of potential regulatory molecules of human SSC. Examining the testis scRNA data from two studies and performing histological validation showed that ASB9 is a molecule predominantly expressed in human SSC.

ASB9 is a SOCS protein family member, which is E3 ubiquitin ligases [37]. Within this family, the six-ankyrin repeat domain-containing ASBs (ASB9, ASB5, ASB13, and ASB11) are a discrete group due to their evolutionary antiquity and a high degree of conservation [44]. ASB9 is involved in the degradation of multiple proteins, such as CKB [37], CKM [39], CKMT1B [40], and HIF1AN [38]. Creatine kinase (CK), an evolutionarily conserved enzyme, is important for the regulation and maintenance of cellular energy stores in tissues with high and rapidly changing energy demands, including cardiac and skeletal muscles as well as the brain [37]. Consequently, ASB9 negatively regulates cell proliferation. ASB9 inhibits ovarian granulosa cell proliferation and promotes differentiation into luteal cells via MAPK signaling [45]. ASB9 affects hepatocarcinogenesis and prognosis by regulating the levels of MtCK [46]. ASB9 negatively regulates the proliferation and invasion of colorectal cancer cells [47]. *Asb9* mRNA has been found to be highly elevated in mouse Pachytene spermatocytes and spermatids, as opposed to spermatogonia [48]. We hypothesized that a mouse model was required further to investigate the function of Asb9 in the mouse testis. This study only explored Asb9 expression at mRNA levels instead of protein levels and lacked important functional validation experiments. ASB9 may aid in elucidating human and mouse species differences in SSCs and spermatogenesis.

HIF1AN is an asparaginyl hydroxylase enzyme that regulates transcriptional activities of hypoxia-inducible factor 1 (HIF1) [49]. HIF-1 is vital in cellular and systemic oxygen homeostasis. HIF-1α regulates the transcription of a variety of genes [50], including erythropoietin, vascular endothelial growth factor, glucose transporters, and glycolytic enzymes, to increase oxygen delivery or promote hypoxia metabolism [51–53]. Glycolysis is the primary mechanism of energy metabolism in SSCs, and it can promote self-renewal in mouse SSCs [54, 55]. Considering these findings, it merits further investigation as to whether ASB9-mediated HIF1AN degradation has other effects on HIF1 signaling and glycolysis. In our study, ASB9 modulates the proliferation of SSC line via HIF1AN in vitro. However, there are limitations to our data. Our experiments are based on human cell lines cultured in vitro, human experiments cannot be performed due to ethical constraints, and evidence from in vivo experiments is lacking. In future studies, we will explore the effect of ASB9 on spermatogenesis by constructing gene conditional knockout mice, and, at the same time, mouse SSC transplantation was used to examine the roles of ASB9 on SSC in vivo, especially the effect on the capacity of SSC to re-establish a complete reproductive lineage.

Our study identified a distinct localization of ASB9 in human SSCs and confirmed its function at the cellular level, but these results are limited. Although ASB9 is predominantly expressed in human SSCs, it is also present in small amounts in differentiating spermatogonia, and its role during differentiation is unknown. This still needs to be confirmed by mouse models and SSC transplantation experiments. In our experiments, we only validated some of the known ASB9 substrates; hence, further investigation is required to determine if there are additional ASB9 regulatory pathways that have not yet been identified. CKB is a typical substrate of ASB9 [37], and up-regulation of ASB9 caused its decrease in SSC line. However, there was no direct effect detected between them, and this result varied from most previously published studies. Although it has also been reported that ASB9 does not interact with CKB in ovarian granulosa cells [56], as an E3 ubiquitin ligase, it is likely that its range of substrate binding is limited and determined by substrate properties like a covalent modification or protein–protein interaction. We cannot exclude that ASB9 may also function by degrading other substrates. It may be best to explore all the interacting proteins of ASB9 using immunoprecipitation and protein mass spectrometry experiments. However, these experiments are currently challenging for us, and we will explore them more in future studies.

We investigated the roles of ASB9 in human SSC line at the cellular level and examined its association with NOA. The results revealed that the overall level of ASB9 protein was significantly decreased in Spg MA samples, whereas the decrease in the percentage of ASB9-positive cells was not significantly different. This may be because, despite the downregulation of ASB9 in individual cells, the cell may still be considered positive. Concurrently, we also need to increase the number of samples to confirm this result. In addition, in vivo experiments are lacking to demonstrate the effects of ASB9 on SSC self-renewal and spermatogenesis. Exome sequencing will be performed on NOA patients to search for potential causative mutation sites in ASB9, and ASB9 knockout mouse models will be constructed to investigate the role of ASB9 in spermatogenesis.

## Conclusion

Overall, we reported for the first time that ASB9 is predominantly expressed in human SSCs and investigated the functions and mechanisms of ASB9 in SSC fate decisions. We also examined the relationship between ASB9 and NOA. Our study provides fresh insights into the developmental regulation of SSCs in humans, as well as novel targets for the treatment of male infertility and the development of contraceptives.

## Supplementary Information

**Additional file 1: Table S1 **Antibodies applied in Western blots, immunofluorescence and immunoprecipitation. **Figure S1. **Genetic maps of expression plasmid inserts. (A) The plasmid map of pCMV3-ASB9-Flag. (B) The plasmid map of pCMV3-HIF1AN-Flag. **Figure S2. **HE staining of testicular tissue in eight patients with azoospermia. According to the Johnsen scoring method, the spermatogenic status of eight samples was assessed as normal (A and B), Spg MA (C and D), Spc MA (E and F) and HS (G and H). Scale bar, 50μm.

## Data Availability

All data are available from the corresponding author upon reasonable request.

## Abbreviations

SSC: Spermatogonial stem cell
NOA: Non-obstructive azoospermia
OA: Obstructive azoospermia
ASB9: Ankyrin-repeat and SOCS box-containing protein 9
GDNF: Glial cell line-derived neurotrophic factor
FGF2: Fibroblast growth factor 2
UMAP: Uniform manifold approximation and projection
PBS: Phosphate buffered saline
PFA: Paraformaldehyde
Spg: Spermatogonia
Spc: Spermatocyte
HS: Hypospermatogenesis
MA: Maturation arrest
