# Supplementary material for: Ubiquitin protein E3 ligase ASB9 suppresses proliferation and promotes apoptosis in human spermatogonial stem cell line by inducing HIF1AN degradation
Source: Biol Res. 2023 Jan 23;56:4. doi: 10.1186/s40659-023-00413-w (PMC9869568; doi:10.1186/s40659-023-00413-w)
Supplement: Supplementary file 1 — Additional file 1: Table S1 Antibodies applied in Western blots, immunofluorescence and immunoprecipitation. Figure S1. Genetic maps of expression plasmid inserts. (A) The plasmid map of pCMV3-ASB9-Flag. (B) The plasmid map of pCMV3-HIF1AN-Flag. Figure S2. HE staining of testicular tissue in eight patients with azoospermia. According to the Johnsen scoring method, the spermatogenic status of eight samples was assessed as normal (A and B), Spg MA (C and D), Spc MA (E and F) and HS (G and H). Scale bar, 50μm. [file 40659_2023_413_MOESM1_ESM.docx]

**Additional file**

**Ubiquitin protein E3 ligase ASB9 affects the proliferation and apoptosis of human spermatogonial stem cells by mediating the degradation of HIF1AN**

**Additional file Data:**

**1 Table**

**2 Figures**

**Additional fileTables**

**Table S1 Antibodies applied in Western blots, immunofluorescence and immunoprecipitation**

| Antibodies | Source | Dilution |
| --- | --- | --- |
| ***Western blot*** |  |  |
| ASB9 | SinoBiological cat#203710-T42 | 1:1000 |
| PLZF | SantaCruz cat#sc-28319 | 1:1000 |
| CCNE1 | Abcam cat#ab33911 | 1:1000 |
| PCNA | Abcam cat#ab29 | 1:500 |
| THY1 | Abcam cat#ab92574 | 1:1000 |
| ACTB | Cwbio cat#CW0096 | 1:2000 |
| CKB | SinoBiological cat# 14415-T52 | 1:1000 |
| HIF1AN | Abcam cat#ab92498 | 1:1000 |
| ***Immunofluorescence*** |  |  |
| ASB9 | SinoBiological cat#203710-T42 | 1:200 |
| UCHL1 | Abcam cat#ab8189 | 1:50 |
| GFRα1 | R&D cat#AF560 | 1:25 |
| PCNA | Abcam cat#ab29 | 1:50 |
| KIT | R&D cat#AF332 | 1:25 |
| ***Immunoprecipitation*** |  |  |
| FLAG | Abcam cat#ab205606 | 1:25 |
| ***Secondary Antibody*** |  |  |
| Goat Anti-Mouse IgG, HRP Conjugated | Cwbio cat#0102 | 1:2000 |
| Goat Anti-Rabbit IgG, HRP Conjugated | Cwbio cat#0103 | 1:2000 |
| Donkey anti-Rabbit IgG,Alexa Fluor 488 | Thermo Fisher Scientific cat#A21206 | 1:1000 |
| Donkey anti-Mouse IgG,Alexa Fluor 594 | Thermo Fisher Scientific cat#A21203 | 1:1000 |
| Donkey anti-Goat IgG,Alexa Fluor 594 | Thermo Fisher Scientific cat#A11058 | 1:1000 |

**
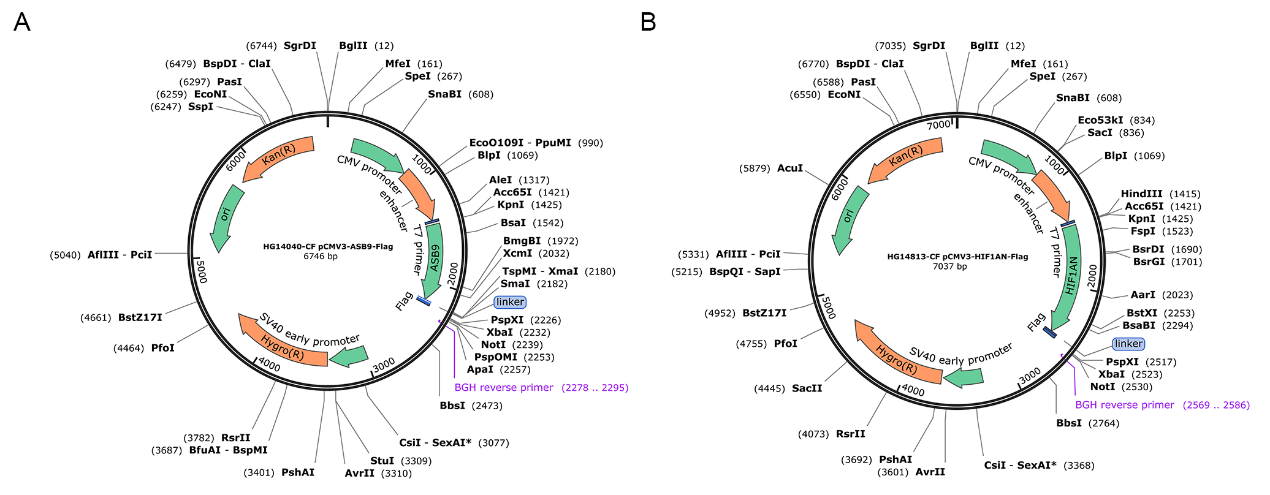
**

**Figure S1. Genetic maps of expression plasmid inserts.** (A) The plasmid map of pCMV3-ASB9-Flag. (B) The plasmid map of pCMV3-HIF1AN-Flag.


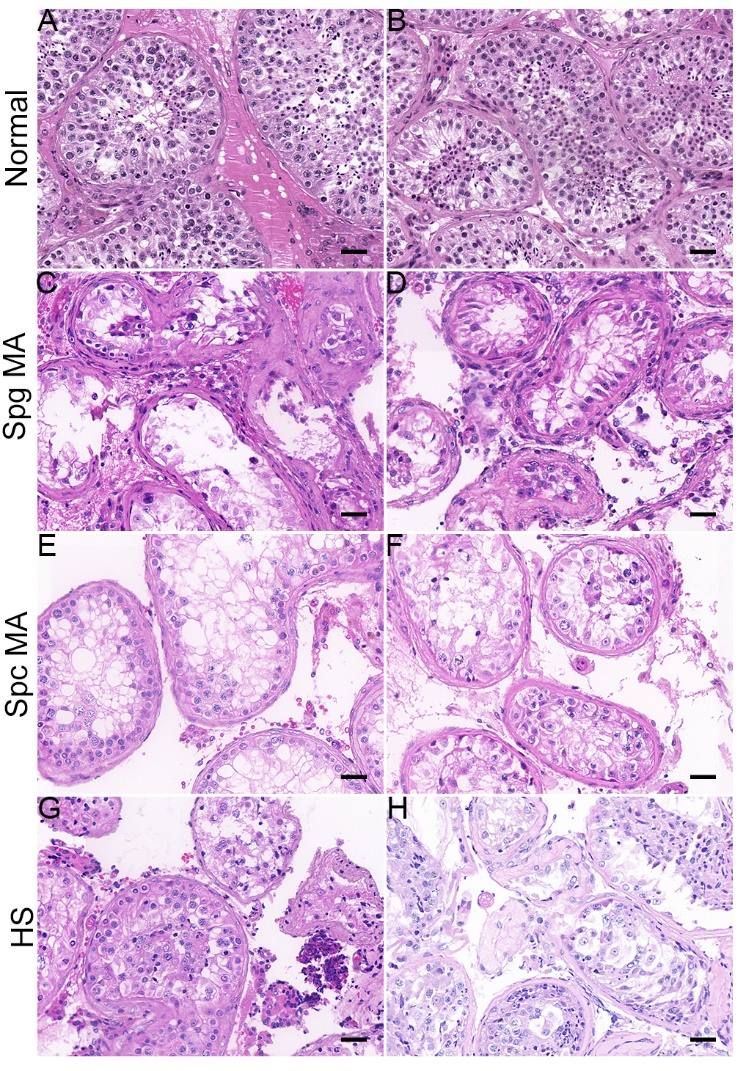


**Figure S2. HE staining of testicular tissue in eight patients with azoospermia**. According to the Johnsen scoring method, the spermatogenic status of eight samples was assessed as normal (A and B), Spg MA (C and D), Spc MA (E and F) and HS (G and H). Scale bar, 50μm.
